# A longitudinal assessment of alcohol intake and incident depression: the SUN project

**DOI:** 10.1186/1471-2458-12-954

**Published:** 2012-11-07

**Authors:** Alfredo Gea, Miguel A Martinez-Gonzalez, Estefania Toledo, Almudena Sanchez-Villegas, Maira Bes-Rastrollo, Jorge M Nuñez-Cordoba, Carmen Sayon-Orea, Juan J Beunza

**Affiliations:** 1 Department of Preventive Medicine and Public Health, University of Navarra, Irunlarrea 1, 31008, Pamplona, Spain; 2 Department of Nutrition, HSPH, Boston, USA; 3 University of Las Palmas de Gran Canaria, Las Palmas de Gran Canaria, Spain

**Keywords:** Alcohol intake, Depression, Cohort analysis, Mediterranean population

## Abstract

**Background:**

Longitudinal studies assessing the long-term association between alcohol intake and depression are scarce. The type of beverage may also be important. Therefore we aimed to prospectively evaluate the influence of alcohol intake on incident depression in a Mediterranean cohort.

**Methods:**

We assessed 13,619 university graduates (mean age: 38 years, 42% men) participating in a Spanish prospective epidemiological cohort (the SUN Project), initially free of depression. They were recruited between 1999–2008 and biennially followed-up during 2001–2010. At baseline, a 136-item validated food–frequency questionnaire was used to assess alcohol intake. Wine was the preferred beverage. Participants were classified as incident cases of depression if they reported a new clinical diagnosis of depression by a physician and/or initiated the use of antidepressant drugs. Cox regression and restricted cubic splines analyses were performed over 82,926 person-years.

**Results:**

Only among women, an U-shaped relationship between total alcohol intake and depression risk was found (*P*=0.01). Moderate alcohol intake (5–15 g/day) was associated with lower risk (Hazard Ratio: 0.62; 95% Confidence Interval: 0.43-0.89). No association was apparent for higher intakes of alcohol or for any specific type of alcoholic beverage.

**Conclusions:**

Moderate alcohol intake might protect against depression among women. Further confirmatory studies are needed.

## Background

Unipolar depressive disorder is the most prevalent mental disease in the world and it is increasing steadily [1]. The prevalence of major depression may reach up to 21% in some populations [2]. Depression is the third leading cause of global disease burden measured in Disability Adjusted Life Years (DALYs), and the first one measured in Years Lost to Disability [3]. If nothing is done, depression will become the first leading cause of global disease burden (DALYs) in 2030 [3]. Besides, 2.25 million deaths per year in the world may be attributed to alcohol intake, even after subtracting the beneficial effects of moderate alcohol intake on the development of cardiovascular disease [4]. In addition, alcohol intake is the 8^th^ global death risk factor and the 3^rd^ risk factor for disease and disability measured in DALYs [4]. Some cross-sectional studies have shown high rates of comorbidity of depression and Alcohol Use Disorders (AUD), which includes alcohol dependence and alcohol abuse [5,6]. Although some cohort and case–control studies have investigated this relationship, the longitudinal association between the quantity of alcohol intake and clinically diagnosed incident depression has not been assessed so far. Moreover reverse causation is a very important threat for the validity of cross-sectional or short-term follow-up studies. Besides, alcohol intake enormously varies among different populations regarding the type of alcoholic beverage, quantity and drinking pattern. We aimed to prospectively evaluate the long-term influence of alcohol intake on the development of depression during a 10 years follow-up in a Mediterranean cohort, where red wine was the most consumed alcoholic beverage.

## Methods

### Subjects

**Figure 1:**
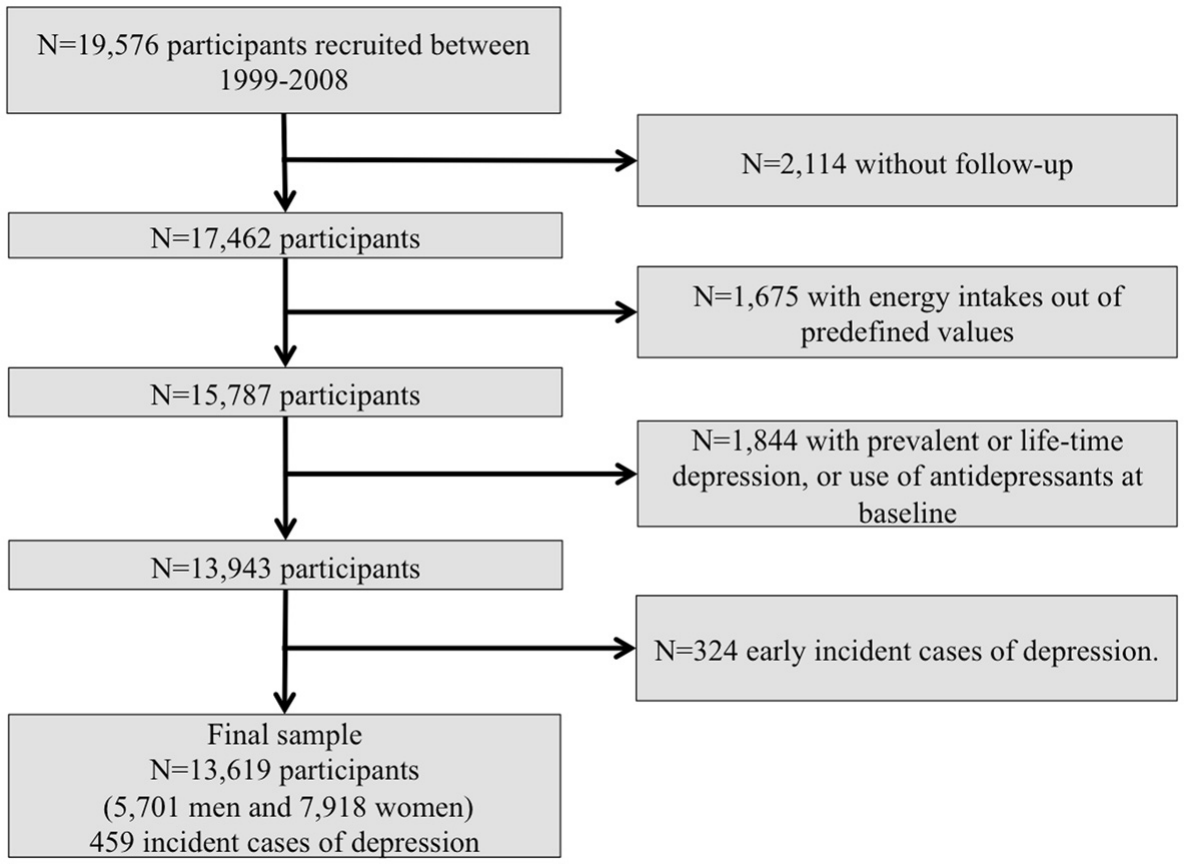
Flow chart of participants: the SUN project.

The “Seguimiento Universidad de Navarra” (SUN) Project is a prospective dynamic cohort, which started in 1999, following the model of the Nurses’ Health Study and the Health Professionals Follow-up Study [7]. Details of the design and methods of this cohort have been described elsewhere [8,9]. Biennial mailed questionnaires were used to obtain updated information from participants. Up to February 2008, 19,576 subjects were recruited. Of these, 17,462 were successfully followed-up (at least one follow-up questionnaire), achieving a retention rate of 89.2%. Subjects with energy intake out of predefined limits (800 Kcal/day or 4,000 Kcal/day in men and 500 Kcal/day or 3,500 Kcal/day in women) [10] were excluded (n=1,675). Other 1,844 subjects who reported prevalent, personal history of previous depression, or use of antidepressants at baseline, and 324 subjects who reported incident depression in the first follow-up questionnaire, up to 2 years of follow-up, were also excluded. The exclusion of incident cases at the second year of follow-up is discussed below. Finally 13,619 participants were included in the analyses (Figure 1). The study was approved by the Institutional Review Board of the University of Navarra. Informed consent was implied by the voluntary completion of the baseline questionnaire.

### Exposure assessment

Alcohol intake was assessed at baseline with a semi-quantitative food-frequency questionnaire (FFQ) that included questions on alcoholic beverage consumption during the past year (red wine, other wines, beer, and spirits). In the validation study for this questionnaire, the correlation coefficient for alcohol intake between the FFQ and four food records was 0.90 [11].

Participants were divided into four groups according to their baseline alcohol intake: abstainers, those who reported drinking less than 10 g/day of alcohol, those with an intake ranging from 10 and 25 g/day and the fourth group with an intake higher than 25 g/day. These groups were reclassified according to the results of the spline in 4 new categories: abstainers, less than 5 g/day, between 5 and 15 g/day, and more than 15 g/day.

In the stratified analysis by type of beverage, alcohol intake was considered as a continuous variable, and adjustment for total alcohol intake was performed through the residuals method [12].

### Outcome assessment

Incident cases of depression were defined as a positive answer by participants in any of the follow-up questionnaires (4^th^, 6^th^, 8^th^, or 10^th^ year) to the question ‘Have you ever been diagnosed of depression by a medical doctor?’ or a positive report after 4 or more years of follow-up habitual use of antidepressant drugs. We excluded the early cases (depression diagnosis made or antidepressant use reported within the first 2 years of follow-up), to avoid reverse causation bias. Antidepressant use was ascertained through an open question in which the participants reported their habitual medication use. This definition of incident depression was validated by a Psychiatrist in a sub-sample of our cohort using the Structured Clinical Interview for DSM-IV (SCID-I) as a gold standard, obtaining a specificity of 0.96; a percentage of confirmed depression of 74% and a percentage of confirmed non-depression of 81% [13].

### Covariate assessment

We obtained the information about medical, socio-demographic, anthropometric, and lifestyle variables from the baseline questionnaire. Physical activity was assessed through a validated physical activity questionnaire [14]. Adherence to the Mediterranean Dietary Pattern (MDP) was evaluated combining 8 items (fruits and nuts, vegetables, fish, legumes, cereals, dairy products, meat and meat products, and the ratio Monounsaturated Fatty Acids/Saturated Fatty Acids) according to the score proposed by Trichopoulou et al. [15], but excluding alcohol intake, that was not taken into account to build the MDP in order to avoid overlapping with our main exposure.

### Statistical analysis

Cox regression models were used to assess the relationship between the four categories of alcohol intake and the incidence of depression. Hazard ratios (HR) and their 95% confidence intervals were calculated using the abstainers group as the reference category. The Cox model included age as the underlying time variable. Birth date was used as the origin variable. Entry time was defined as date at recruitment. Exit time was defined as date at diagnosis of depression for cases and for participants who did not develop depression as date when completing the last follow-up questionnaire or as age at death (whichever occurred first). For the multiple-adjusted model, the following potential confounders were considered: smoking, physical activity (MET-h/week), total energy intake (Kcal/day), body mass index (kg/m^2^), adherence to the MDP, marital status, and employment status. We evaluated the interaction between sex and alcohol intake by introducing an interaction term in the model. Although there was not an effect modification by sex, the analyses were also conducted separately for men and women. We conducted sensitivity analyses re-running all the models after: 1) including also early cases (up to the first two years of follow-up), 2) excluding late cases of depression (after 10 years), 3) excluding prevalent cases of other psychiatric diseases (insomnia, schizophrenia, anxiety, anorexia and bulimia, stress, obsessive compulsive disorder, bipolar disorder, phobias) at baseline, 4) excluding prevalent cases of cancer or cardiovascular disease (angina pectoris, coronary surgery including bypass, coronary angioplasty, stroke including thrombosis, embolism, and cerebral haemorrhage, paroxysmal tachycardia, atrial fibrillation, aortic aneurysm, heart failure, pulmonary embolism, peripheral venous thrombosis, intermittent claudication, and all types of cancer) at baseline, 5) depurating the abstainers group (excluding former drinkers and participants who do not drink due to medical causes), 6) excluding participants under anxiolytic drugs or other psychiatric medication at baseline, 7–9) excluding participants under anxiolytic, antipsychotic, or antiepileptic or anticonvulsant medication during the follow-up period.

Finally, we evaluated the potential non-linear association between alcohol intake and incident depression non-parametrically calculating the HR and 95% CI with restricted cubic splines [16], stratified by sex. Tests for non-linearity used the likelihood ratio test comparing the model with only the linear term to the model with the linear and the cubic spline terms. The results were adjusted for the same potential confounding factors as the main Cox regression analysis.

We also conducted spline analysis on alcohol independently for each alcoholic beverage (beer, wine, spirits) as a continuous variable. All *P*-values were two-tailed and *P*<0.05 was considered significant.

## Results

The main characteristics of the 13,619 participants (5,701 males and 7,918 females) categorized according to their alcohol intake are presented in Table 1. High alcohol intake (>25 g/day) was associated with being male (86% were male), older (mean age was 46 years), and with higher BMI (mean BMI 26 Kg/m^2^).

**Table 1:** Baseline characteristics according to categories of alcohol intake stratified by sex: mean and SD, or %

	**MALES**				**FEMALES**			
**Alcohol intake categories (g/day)**	**0**	**<10**	**10-25**	**>25**	**0**	**<10**	**10-25**	**>25**
N	628	3,017	1,462	594	2,141	4,904	778	95
Age (years)	43 (15)	42 (13)	43 (12)	49 (11)	36 (11)	34 (10)	37 (11)	42 (9)
BMI (kg/m^2^)	25 (3)	25 (3)	26 (3)	26 (3)	22 (3)	22 (3)	22 (3)	22 (3)
Physical activity (MET-h/week)	28 (35)	25 (25)	25 (25)	24 (22)	18 (20)	18 (19)	20 (19)	20 (18)
Mediterranean dietary pattern	4 (2)	4 (2)	4 (2)	4 (2)	4 (2)	4 (2)	4 (2)	4 (2)
Current smokers (%)	13	16	26	27	15	25	35	30
Ex-smokers (%)	29	35	37	53	20	27	34	48
Marital Status (% married)	59	63	64	80	49	43	39	56
Living alone (%)	5	5	6	8	5	7	10	14
Unemployment (%)	3	2	2	2	5	6	4	2
***Alcohol intake source***								
Alcohol (g/day)	0 (0)	5 (3)	16 (4)	41 (19)	0 (0)	3 (3)	15 (4)	33 (9)
Red Wine (g/day)	0 (0)	1 (2)	6 (5)	19 (14)	0 (0)	1 (2)	5 (5)	16 (13)
All wine (g/day)	0 (0)	2 (2)	7 (6)	23 (16)	0 (0)	1 (2)	6 (6)	17 (14)
Beer (g/day)	0 (0)	2 (2)	5 (4)	11 (14)	0 (0)	1 (2)	5 (4)	12 (12)
Spirits (g/day)	0 (0)	1 (1)	4 (4)	7 (12)	0 (0)	1 (1)	3 (4)	4 (7)

The SUN Project 1999–2010.

A total of 459 incident cases of depression were identified during the follow-up period, which summed up 82,926 person-years.

The Cox regression analysis considering all the participants in the study categorized in four groups at cut-off points (>0, 10, and 25 g/day) showed an inverse association that was not statistically significant (Table 2).

**Table 2:** HR and 95% CI for incident depression according to categories of daily alcohol intake in the overall sample and stratified by sex

***Alcohol intake categories (g/day)***	**0**	**<10**	**10-25**	**>25**
	OVERALL SAMPLE			
Cases/Person-years	114/16,921	255/48,206	68/13,711	22/4,088
Age and sex adjusted	1 (Ref.)	0.84 (0.67-1.05)	0.83 (0.61-1.14)	0.91 (0.56-1.46)
Multiple-adjusted model ^a^	1 (Ref.)	0.83 (0.66-1.04)	0.81 (0.59-1.11)	0.86 (0.53-1.39)
Interaction between sex and categories of alcohol intake	*P*=0.644			
	MALES			
Cases/Person-years	22/3,814	76/18,654	41/9,130	17/3,513
Age adjusted	1 (Ref.)	0.68 (0.42-1.10)	0.75 (0.45-1.26)	0.77 (0.40-1.45)
Multiple-adjusted model ^b^	1 (Ref.)	0.69 (0.43-1.11)	0.76 (0.45-1.29)	0.76 (0.40-1.47)
	FEMALES			
Cases/Person-years	92/13,107	179/29,552	27/4,582	5/575
Age adjusted	1 (Ref.)	0.89 (0.69-1.14)	0.81 (0.52-1.24)	1.06 (0.43-2.61)
Multiple-adjusted model ^b^	1 (Ref.)	0.87 (0.67-1.13)	0.78 (0.50-1.21)	1.06 (0.43-2.63)

a. Adjusted for age, sex, smoking, physical activity (MET-h/week), total energy intake (Kcal/day), baseline body mass index (kg/m^2^), adherence to the MDP, marital status, and employment status.

b. Excluding sex from the previous multiple-adjusted model.

The SUN Project 1999–2010.

We did not find any effect modification of alcohol according to the sex of the participants (*P*=0.644). Although the interaction was non-significant, we fitted again the Cox regression stratified by sex. The reasons for this sub-groups analysis are discussed below. Stratified analysis showed no association between alcohol intake and incident depression (Table 2).

We carried out multiple sensitivity analyses to rule out possible sources of bias in the estimation of the association between alcohol intake and depression (data not shown; available on request). We repeated the analyses after excluding prevalent and life-time cases of psychiatric diseases at baseline (n=85), after excluding prevalent or life-time cases of cardiovascular disease or cancer at baseline (n=410), after excluding later incident cases of depression reported after 10 years of follow-up (n=8), after depurating the abstainers group excluding former drinkers and participants who do not drink due to health reasons (n=227), after including earlier incident cases (n=324), after excluding participants under anxiolitic drugs or other psychiatric medication at baseline (n=324), and after excluding participants under anxiolytic, antipsychotic, or antiepileptic or anticonvulsant medication during the follow-up period (n=455, n=114, n=36 respectively). The associations between alcohol intake and depression observed in all these sensitivity analyses were similar to those obtained in the main analysis.

**Figure 2:**
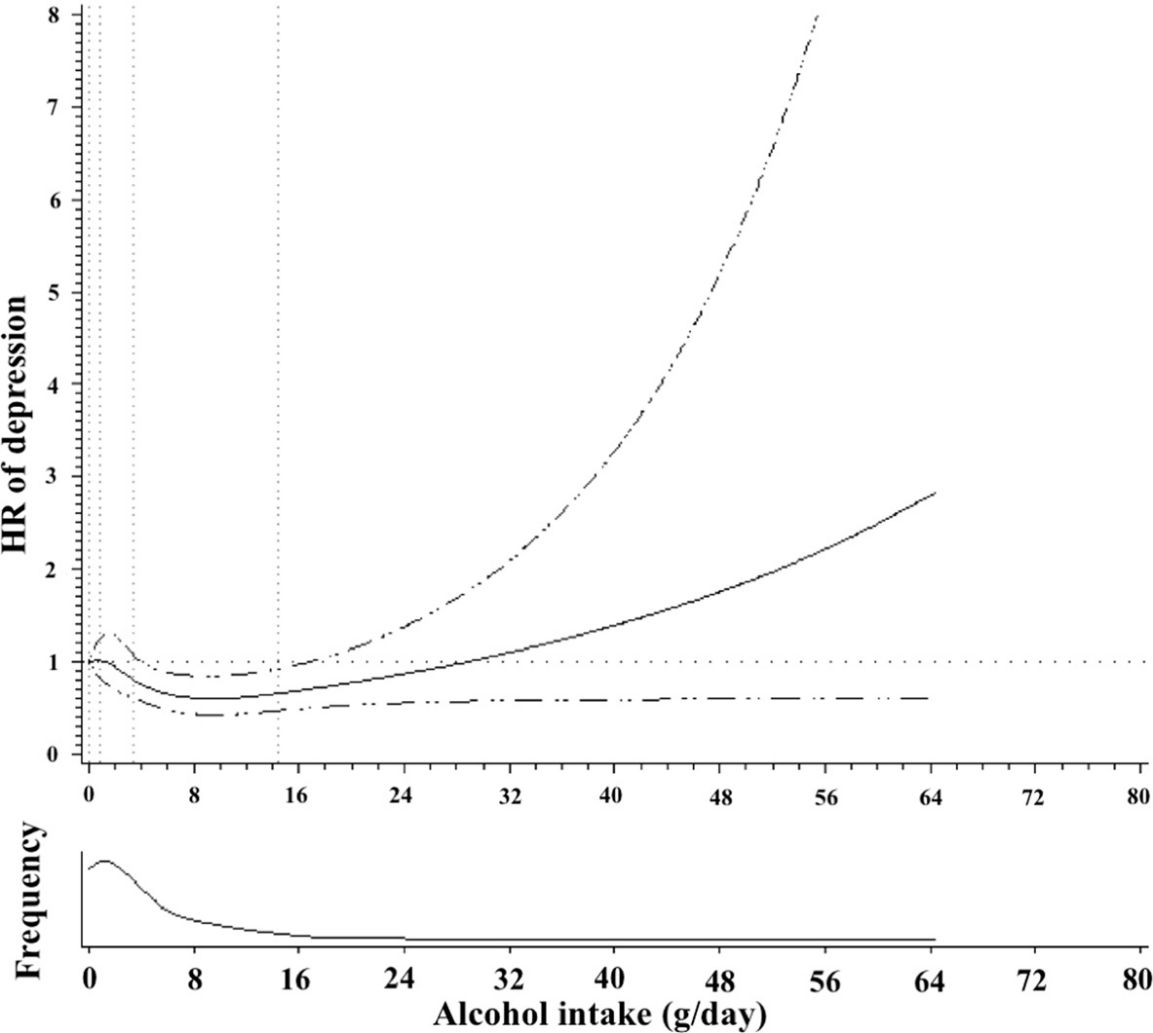
**Association between alcohol intake (g/day) and depression risk among women (HR and 95% CI).** Black line represents the HR and the pointed-lines represent the 95% confidence interval. Adjusted for age, smoking, physical activity (MET-h/week), total energy intake (Kcal/day), baseline body mass index (kg/m^2^), adherence to the MDP, marital status, and employment status.

To account for a non-linear association, we performed restricted cubic spline analysis adjusted for the same potential confounding factors as the main Cox regression analysis. Among men, we found no statistically significant linear or non-linear association between alcohol intake and incident depression (*P*=0.21 and *P*=0.49 respectively). We found no relationship for any type of specific alcoholic beverage nor for total alcohol intake among men. Among women, we found a significant inverse relationship between total alcohol intake and the incidence of depression. This relationship was statistically significant for the non-linear association (*P*=0.01). The U-shaped association that was found is graphically represented in Figure 2. Women with an alcohol intake ranging from 5 to 15 g/day showed a significantly lower risk of depression (the HR and the 95% CI were below the null value). In order to obtain estimates for specific categories of alcohol intake, we fitted another Cox regression analysis adapting the building of groups of alcohol intake to the shape of the dose–response curve obtained using the restricted cubic spline model: abstainers, <5 g/day, 5–15 g/day, and >15 g/day. This resulted in a HR and 95% CI of 0.62 (0.43-0.89) for the third category (5–15 g/day) as compared to abstainers but no association was found for the rest of categories (Table 3).

**Table 3:** HR and 95% CI for incident depression according to categories of daily alcohol intake adapted to the spline results

***Alcohol intake categories (g/day)***	**0**	**<5**	**5-15**	**>15**
	OVERALL SAMPLE			
Cases/Person-years	114/16,921	204/33,011	95/23,242	46/9,753
Age and sex adjusted	1 (Ref.)	0.96 (0.76-1.21)	0.66 (0.49-0.87)	0.77 (0.53-1.10)
Multiple-adjusted model ^a^	1 (Ref.)	0.94 (0.75-1.19)	0.65 (0.49-0.86)	0.73 (0.50-1.06)
	MALES			
Cases/Person-years	22/3,814	53/10,911	49/12,776	32/7,610
Age adjusted	1 (Ref.)	0.82 (0.49-1.34)	0.64 (0.39-1.07)	0.68 (0.39-1.17)
Multiple-adjusted model ^b^	1 (Ref.)	0.82 (0.50-1.36)	0.64 (0.39-1.07)	0.68 (0.39-1.19)
	FEMALES			
Cases/Person-years	88/12,629	136/19,523	59/12,578	12/1,706
Age adjusted	1 (Ref.)	1.01 (0.78-1.31)	0.62 (0.44-0.89)	0.86 (0.49-1.51)
Multiple-adjusted model ^b^	1 (Ref.)	0.97 (0.75-1.27)	0.62 (0.43-0.89)	0.84 (0.47-1.48)

a. Adjusted for age, sex, smoking, physical activity (MET-h/week), total energy intake (Kcal/day), baseline body mass index (kg/m^2^), adherence to the MDP, marital status, and employment status.

b. Excluding sex from the previous multiple-adjusted model.

The SUN Project 1999–2010.

No associations were found for the consumption of specific beverage types and depression risk in the overall sample neither in the analyses separated by sex (data not shown).

## Discussion

The results of this prospective study suggest that whereas moderate alcohol intake (5–15 g/day) may protect women against the development of depression, higher amounts of alcohol intake may not confer any protection against depression.

Many studies have investigated the relationship between exposure to alcohol and depressive status. However, most of them have focused on subjects with AUD more than in subjects with alcohol intake levels in a lower range. Some other studies [17,18] have longitudinally investigated the relationship between alcohol dependence or alcohol abuse, but not categories of alcohol intake, and major depression. In fact, a recent review [19] reported a positive association between AUD and major depression.

However, longitudinal studies focusing on a normal range of alcohol intake even if they are follow-up studies, may still be affected by reverse causation bias because the follow-up is relatively short (only 1–2 years), or because they have not excluded prevalent cases of depression, or have assessed depression only on a narrow window of time during follow-up. Moreover no Mediterranean cohort, with a higher red wine consumption, has ever assessed this association. Therefore the differences in the assessment of exposure and/or events, or in the type of patients evaluated make the comparison with these studies difficult. A study from the Health and Retirement Study [20] found an association between problematic alcohol intake and depression in a cohort of men aged over 50, but no association was found with non-problematic alcohol intake. Biennially for 6 years, alcohol intake was assessed through questionnaires, and incidence of depression was assessed using a symptoms scale with a cut-point. However, 63% of the participants were classified as problematic alcohol drinkers even when they reported drinking less than 1 drink per week. In addition, some cases of depression might be underestimated in that study since they only evaluated self-reported cases of depression during the last 2 years of the study, but not those occurring in the first 4 years of follow-up. Finally, that study only included men since the exposure was problematic drinking, which is much more prevalent among men. Another study examined how alcohol use predicts changes in psychological symptoms among 393 adolescents followed up for 18 months [21] and found that initial levels of alcohol use did not predict changes in depression. Inclusion criteria for that study were to score one standard deviation above the school mean on one of the four subscales of the Substance use Risk Personality Scale: hopelessness, anxiety, impulsivity, and sensation seeking. In addition, depression was assessed through the Brief Symptom Inventory, not through a doctor made diagnosis. Finally, previous or prevalent cases of depression were not excluded since diagnosis of depression as such was not assessed. Paljärvi et al. [22] performed a two-wave 5-years follow-up study to determine which aspect of drinking pattern would be the best predictor for depressive symptoms. Self-reported depressive symptoms were measured with a questionnaire at baseline and at year-5. They found that binge-drinking and also higher categories of alcohol intake were directly associated with depressive symptoms. These results are in contrast with our findings, however Paljärvi el al. excluded abstainers from their analyses, and used the moderate alcohol consumers as the reference category. Skogen et al. [23] found an U-shaped association between alcohol intake and both anxiety and depression. They distinguished between the possible former drinkers and abstainers. However, these authors acknowledged that the direction of causality was not clear. Another study evaluated the influence of alcohol dependence on the first incident depressive episode in a 1-year follow-up [24]. Both disorders were investigated using Diagnostic Interview Schedule, according to DSM-III. Prevalent and previous cases of depression were excluded. Multivariate logistic regression was conducted separately for men and women, obtaining a stronger positive association for women. These results are not comparable with ours because we did not assess alcohol dependence as exposure.

Some case–control studies have investigated the influence of alcohol intake on the development of depression [25-27]. For example, an study conducted by Armenian et al. [27] after an earthquake found a protective association for alcohol intake in cases of depression without comorbidity of other psychiatric disorders: OR (95% CI) =0.6 (0.3-0.9). The post-traumatic situation makes our results not comparable with the results of this study. Two other studies found contradictory results [25,26]. However, these case–control studies may also be affected by reverse causality bias.

Finally, some cross-sectional studies have found high comorbidity between both conditions [4]; however, their cross-sectional nature is an obstacle to assess causality, especially when it is well known that depression is a risk factor for AUD [17,18].

Our results suggest an U-shaped association between alcohol intake and the development of depression, only for women. The non-significant association for heavy alcohol intake may be due to the very low average consumption and the scarcity of heavy drinkers in our cohort. This is consistent with Perreira et al. [20] who did not find any association for men. However, our results are not consistent with Mackie et al. [21] who did not find any association for men or women. These studies, and all cited above, are not generally comparable because of the differences in the alcohol and depression assessments [28]. Moreover, the different results between these studies may be due to the differences in the distribution of alcohol intake between populations. When the population has moderate to low average alcohol intake, the association tends to be inverse or null [20,21] and when the population has heavy alcohol intake, it tends to be positive [20,22]. When assessing the relationship between alcohol intake and the development of depression a careful consideration of the temporal sequence is needed. In order to reduce the risk of falling into reverse causation bias, early incident cases of depression were excluded. These cases may be sub-clinical cases of depression that are likely to increase their alcohol intake as a consequence of their recent depression. On the contrary, alcohol intake information was collected at baseline and some cases of depression were developed after 10 years of follow-up. Considering on the other hand that this late incident cases might not be influenced by alcohol intake at baseline, we excluded these later cases and the magnitude of the association did not change considerably.

The development of depression may be influenced by the presence of other psychiatric disorders [29]. In order to control for this possible bias we conducted the analysis after the exclusion of prevalent and lifetime cases of psychiatric disorders. The association did not change substantially. Other important diseases like cancer [25,30] or cardiovascular disease may lead to the development of depression and also to changes in alcohol intake. After the exclusion of prevalent cases of both kinds of disorders, the association remained similar. Other potentially important issue is the comparison group. Abstainers might be a heterogeneous group. Those who do not drink may have different reasons not to do so. Some of them may be abstainer due to medical advice. Participants declaring being abstainers could have been former drinkers. These facts could bias the results towards a more protective association. However, after excluding former drinkers and those abstainers who did not drink due to medical advice, the association between alcohol intake and the development of depression did not change.

We conducted the analysis separately for men and women although the interaction product-term was not significant. However, previous studies [31,32] found differences between both sexes, therefore we considered convenient to split our sample. Women seem to be more likely to the development of depression [33] and the same alcohol intake affects differently to men and women [34] due to their different body composition, affecting the volume of distribution, or different metabolism.

Some mechanisms have been proposed to explain the deleterious effect of heavy or chronic alcohol drinking on brain function [35]; however, moderate alcohol drinking have a GABAergic effect, acting on GABA_A_ receptors [36], that may prevent or counteract the effects of depression on this system [37].

Our study has some important strengths, such as its prospective design (avoiding reverse causation bias), data collection and data analysis. Other strengths are its large sample size, its high retention rate, the good adjustment for potential confounders, the existence of published validation studies for our methods, the high correlation observed between the FFQ and the food records for alcohol intake in the validation study, and the high educational level of the participants, achieving high quality information and high internal validity. In our analysis, we have carefully considered the reverse causation bias, used validated diagnosis of depression, taken into account other psychiatric disorders, analysed sex-alcohol interaction, excluded other important diseases, and depurated the abstainers group.

A limitation of our study is the use of a self-reported clinical diagnosis of depression. We assume that a low proportion of participants could misreport the diagnosis. Moreover, participants who reported habitual use of antidepressant drugs were also considered incident cases. Research suggests first treatment for depression typically occurs several years following the onset of depression. The median time from onset of depression to first treatment has been reported to be 8 years in the U.S. population [38]. Long lags in first contact for depression treatment have also been reported in other countries [39]. Thus, identifying incident cases of depression by having received a diagnosis or prescribed medication by a medical doctor may result in missed cases. This is especially true for cases recruited later in the study who were followed up for a shorter period of time than case recruited earlier in the study. These missing cases can translate in low sensitivity of our case ascertainment definition. However, our validation study found very high specificity (0.96) for the self-reported diagnosis of depression and theoretically with perfect specificity, non-differential misclassification of disease, due to low sensitivity, will not bias the relative risk estimate [40].

A potential limitation is that few participants were heavy alcohol consumers, which may lead to a lack of statistical power to assess the relationship with depression for high levels of alcohol intake. Another limitation is that we do not have information about illicit drug use. However the SUN cohort participants are highly motivated, responsible and health-conscious who voluntarily agreed to complete long and complicated questionnaires. We expect that the use of illicit drugs will be very low or even non-existent among them.

## Conclusions

Moderate alcohol intake (5–15 g/d) among women might protect them against the development of depression, while high alcohol intake seems to confer no benefit. However, additional cohort studies are needed to confirm our findings.

## Abbreviations

SUN: Seguimiento Universidad de Navarra; HR: Hazard ratio; 95% CI: 95% Confidence interval; DALYs: Disability adjusted life years; AUD: Alcohol use disorders; FFQ: Food frequency questionnaire; SCID-I: Structured Clinical Interview for DSM-IV; MDP: Mediterranean Dietary Pattern; MET: Metabolic equivalent; BMI: Body mass index; OR: Odds ratio; GABA: Gamma aminobutiric acid.

## Competing interest

There is no competing interest. There is no financial arrangement with any food or alcoholic beverages company.

## Authors’ contributions

AG conducted the literature review, participated in the design of the study, conducted the main statistical analyses and prepared the first draft of the manuscript. ET participated in the design of the study, conducted part of the statistical analyses and supervised the methods section. ASV participated in the design of the study, conducted part of the literature review, contributed to the interpretation of findings, the writing of the discussion section, and obtained funding. MBR participated in the design of the study, the interpretation of results, and obtained funding and administrative support. JMNC participated in the interpretation of results. CSO helped with the literature review and with the statistical analyses. JJB directed and supervised the study, participated in the statistical analyses, the interpretation of results, and obtained funding and administrative support. MAMG was the founder and principal investigator of the SUN cohort, supervised all the steps in the statistical analyses and preparation of the manuscript, and obtained funding and administrative support. All authors took care of the critical revision of the manuscript for important intellectual content and approved the final version to be submitted for publication.

## Pre-publication history

The pre-publication history for this paper can be accessed here:

http://www.biomedcentral.com/1471-2458/12/954/prepub
